# Hemocompatibility of Albumin-Modified Magnetic Nanoparticles

**DOI:** 10.3390/ijms252211975

**Published:** 2024-11-07

**Authors:** Indu Sharma, Mehdi Gaffari Sharaf, Aishwarya Pawar, Agatha Milley, Larry D. Unsworth

**Affiliations:** Department of Chemical and Material Engineering, University of Alberta, Edmonton, AB T6G 1H9, Canada; indu2@ualberta.ca (I.S.); mghaffar@ualberta.ca (M.G.S.); aspawar@ualberta.ca (A.P.); amilley@ualberta.ca (A.M.)

**Keywords:** albumin, magnetic nanoparticles, hemodialysis, adsorption, clot kinetics

## Abstract

Kidney failure leads to the accumulation of metabolites in the blood compartment. This build-up of metabolites has been associated with increased mortality and morbidity in these patients; thus, these metabolites are commonly called uremic toxins. The retention of some uremic toxins in the blood results from a strong interaction with serum albumin, preventing their clearance using standard hemodialysis techniques. Adsorbents are considered the next-generation technology for clearing uremic toxins from the blood, and iron oxide magnetic nanoparticles are a promising material due to a high surface area that is easily modified and the ability to remove them from blood with an external magnetic field. Plasma protein adsorption and clot formation kinetics were determined for unmodified and albumin-modified iron oxide magnetic nanoparticles. Albumin was selected because it can bind uremic toxins, and it is commonly used to passivate surfaces. Coatings were formed and characterized using transmission electron microscopy, thermogravimetric analysis, and zeta-potential analysis. Clotting kinetics, total protein assays, and immunoblots were used to analyze the effect surface modification has on protein adsorption events. Unmodified nanoparticles showed rapid clotting and more adsorbed protein compared to albumin-coated iron oxide nanoparticles. Immunoblots show that modified particles showed changes in albumin, protein C, Immunoglobulin G, transferrin, fibrinogen, α1-antitrypsin, vitronectin, plasminogen, prothrombin, and antithrombin levels compared to unmodified controls. The hemocompatibility of adsorbent materials is essential to their clinical application in clearing the blood of uremic toxins.

## 1. Introduction

Kidney failure leads to the retention of metabolites (uremic toxins) in the blood. Some of these uremic toxins (UTXs) have been linked to severe complications [1]. Hemodialysis is a life-saving intervention, but it consumes vast resources while delivering suboptimal outcomes. Namely, hemodialysis struggles to clear mid-sized molecules and protein-bound uremic toxins (PBUTs), leaving patients at risk of chronic inflammation and other severe health outcomes [2,3,4]. There is an urgent need to improve toxin removal from the blood of these patients [5].

The development of advanced materials that can enhance the efficiency and safety of hemodialysis is therefore of paramount importance [6]. Adsorbent-based strategies hold great potential for enhancing the clearance of otherwise difficult-to-remove molecules, like mid-size molecules and PBUTs. The clearance of these compounds could lead to improved clinical outcomes, a better quality of life, and reduced healthcare costs [4,7]. Iron oxide magnetic nanoparticles (MNPs) have emerged as promising candidates for a variety of biomedical applications, including magnetic resonance imaging, targeted drug delivery [8,9,10], and hyperthermia treatment for cancer [11,12]. When biomaterials are introduced into the body, they initiate a host cascade response, starting with protein adsorption and forming a bio-corona. The composition and structure of protein corona play a crucial role in how the immune system and other cells interact with materials, acting as an early indicator of the host’s overall response [13,14,15]. The type, density, and orientation of the proteins within this corona can either activate immune responses, enhance cellular uptake, or signal for the material to be disregarded by the cells. Therefore, understanding these interactions is essential, as they ultimately determine the biocompatibility of materials and how well they integrate with or are rejected by the host system [13,16,17]. With appropriate surface chemistry, nanoparticles have been designed and used experimentally for in vivo applications like tissue repair, immunoassay, and detoxification, as they are generally considered biocompatible and safely biodegradable [18,19].

Modifying nanoparticles with albumin is relatively common, given that its presence as a coating imbues the surface with relatively good hemocompatibility, low immunogenicity, and functional versatility [20,21]. Moreover, albumin coatings inhibit nanoparticle aggregation while minimizing non-specific protein adsorption [22,23]. The albumin layer may also provide a means of binding UTXs as it has been shown, by our lab and others, that albumin can bind PBUTs, like indoxyl sulfate (IS) and p-cresyl sulfate (PCS), indole 3-acetic acid (IAA), hippuric acid, 3-carboxyl-4-methyl-5-propyl-2-furan propanoic acid (CMPF), and phenylacetic acid (PAA) [24,25]. Current knowledge of the mechanisms dictating nanoparticle immune recognition and stability in biological fluids is limited [26]. Further research is needed to clarify these interactions to optimize iron oxide magnetic nanoparticles’ (MNPs) hemocompatibility [27].

It is hypothesized that the surface modification of iron oxide magnetic nanoparticles with albumin will provide a lower adsorbed amount of protein and alter the general adsorbed proteome whilst inhibiting clot formation induced through this adsorbed protein layer. To this end, we covalently bound bovine serum albumin (BSA) on iron oxide magnetic nanoparticles, yielding two different surface densities of albumin. These particles were thoroughly characterized using transmission electron microscopy (TEM), thermogravimetric analysis (TGA), and zeta-potential analysis. Clotting kinetics, total protein assays, and immunoblots were used to analyze the effect surface modification has on protein adsorption events. Understanding the general hemocompatibility of these albumin coatings on iron oxide magnetic nanoparticles (MNPs) lays the foundation for further exploring their use as adsorbents for removing UTXs from blood.

## 2. Results

### 2.1. Particle Size and Coating Thickness

**Figure 1:**
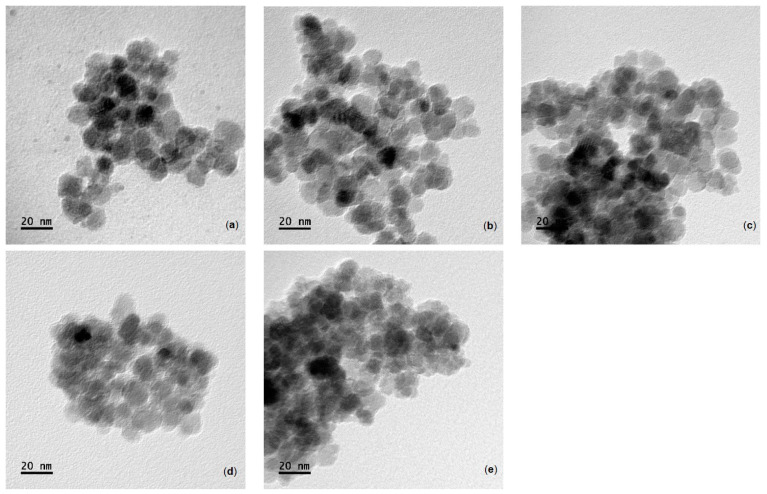
TEM images of (**a**) MNPs-Bare, (**b**) MNPs-APTES, (**c**) MNPs-APTES-GA, (**d**) MNPs-APTES-GA-BSA (0.2), and (**e**) MNPs-APTES-GA-BSA (2).

**Figure 2:**
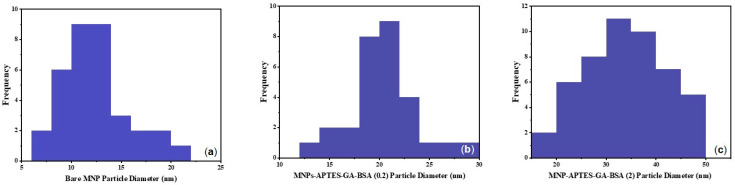
Histogram of the size distribution of (**a**) MNPs-Bare (n = 34), (**b**) MNPs-APTES-GA-BSA (0.2) (n = 29), and (**c**) MNPs-APTES-GA-BSA (2) (n = 47).

Iron oxide magnetic nanoparticles (MNPs), before and after albumin modification, were imaged using transmission electron microscopy (TEM) to find the size, shape, and overall homogeneity (Figure 1). Bare MNPs were relatively uniform and spherical (Figure 1a), with diameters between 8 and 14 nm (ave: 12.4 ± 3.3 nm, n = 35, Figure 2a) [28,29,30,31]. (3-Aminopropyl)triethoxysilane (APTES) and glutaraldehyde (GA) modification induced no change in iron oxide magnetic nanoparticles size or morphology, which was similar to previous reports (Figure 1b,c) [28,31,32]. TEM images clearly showed albumin coating formation (the lighter layer around particle edges) (Figure 1d,e) with an average particle diameter of 20.3 ± 3.6 and 34.12 ± 8.1 nm when formed in solutions with BSA concentrations of 0.2 and 2.0 mg/mL, respectively (Figure 2b,c). Coatings formed at 0.2 mg/mL of albumin showed something similar to an expected monolayer based on the hydrodynamic radius of BSA [33,34]. For 2 mg/mL systems, previous work showed diameters between 27 and 42 nm depending upon the type and size of iron oxide magnetic nanoparticles (MNPs) used along with the strategy involved for albumin attachment [31,35]. It was observed that Alb-MNPs were less affected by a magnetic field compared to unmodified iron oxide magnetic nanoparticles. Iron oxide magnetic nanoparticles (MNPs) modified using a high albumin solution concentration had a globular shape and homogeneous spherical morphology and were observed in larger aggregates. Some excess protein may be present, as evidenced by light and cloudy residue in images, making it harder to determine the thickness of the albumin coating, but thick crosslinked albumin was present. For statistical analysis, Student’s *t*-test was conducted using Origin 2024 for bare, MNPs-APTES-GA-BSA (0.2), and MNPs-APTES-GA-BSA (2). The *p*-values calculated for the bare and MNPs-APTES-GA-BSA (0.2) are lower than 0.0001; for bare and MNPs-APTES-GA-BSA (2), these are also lower than 0.0001; and for MNPs-APTES-GA-BSA (0.2) and MNPs-APTES-GA-BSA (2), the *p* value is lower 0.001. These results indicate that all coating steps showed statistically significant differences between all the groups, suggesting that the BSA coating, at both 0.2 mg/mL and 2 mg/mL concentrations, significantly changed the properties of the MNPs compared to the bare nanoparticles, and there were also significant differences between the BSA coating at both 0.2 mg/mL and 2 mg/mL concentrations.

### 2.2. Relative Mass of Albumin Coatings

**Figure 3:**
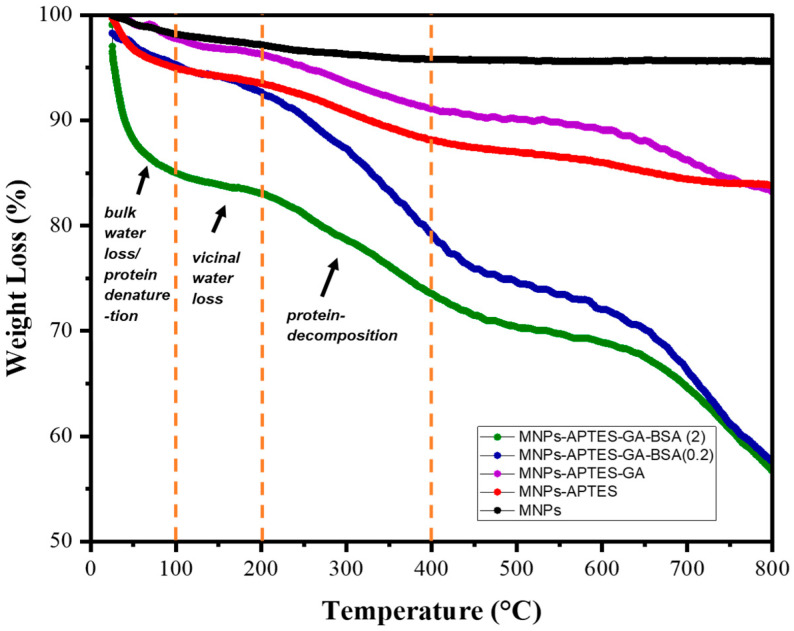
TGA data for MNPs, MNPs-APTES, MNPs-APTES-GA, MNPs-APTES-GA-BSA (0.2), MNPs-APTES-GA-BSA (2). Under nitrogen flow, TGA was conducted with a heating rate of 10 °C/min and a temperature range of 25–800 °C.

Thermogravimetric analysis (TGA) was conducted at every step of the albumin modification process (Figure 3). Mass loss at temperatures <100 °C was largely attributed to the loss of bulk water, and temperatures from 100 to 200 °C were assigned to vicinal water. As expected, unmodified MNPs showed only ~5.5% total weight loss upon heating since MNPs do not degrade within this temperature range and have a lower bulk and vicinal water content [19,36]. An increased percentage of weight loss after each chemical modification step further confirmed a successful reaction. The curve for the APTES-modified sample closely resembled that of the bare MNPs, except for the added weight loss due to the APTES. With the addition of glutaraldehyde, less weight was lost at lower temperatures and more at higher temperatures than APTES, suggesting an enhanced thermal stability of the coating upon glutaraldehyde crosslinking. BSA degradation in coatings on MNPs was shown to be independent of the solution concentration of BSA [37].

According to the literature, weight decreases at temperatures up to 100 °C can be associated with BSA denaturation as well as water loss [36,37]. Furthermore, albumin decomposes in the temperature range of 250–400 °C [37,38]. It was observed that up to 110 °C, a broad peak was present, where an endothermic effect was attributed to BSA denaturation [5,37,38,39]. With increasing BSA concentration from 0.2 to 2 mg/mL, this peak intensifies and shifts towards a slightly higher weight loss. The mass loss between 200 and 380 °C is assigned to the breaking of the C-O and C-C bonds. So, the mass loss in this range was highest for MNPs-APTES-GA-BSA (2), supporting the TEM data showing a higher amount of incorporated albumin for these coatings.

### 2.3. Particle Surface Chemistry Analysis

**Figure 4:**
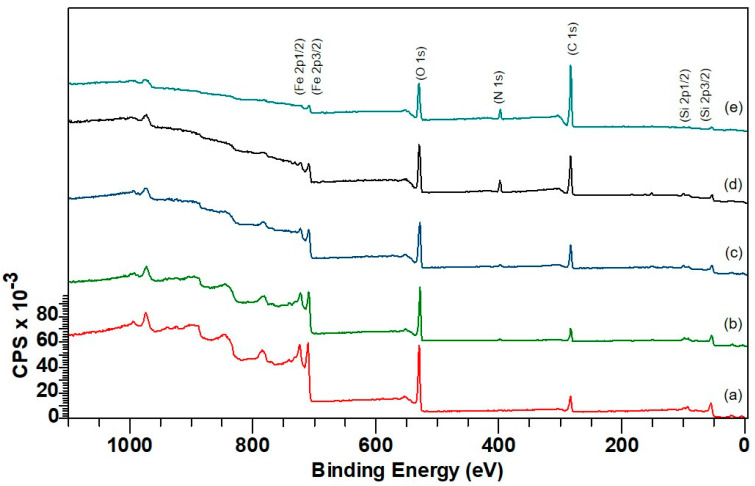
Survey scan of (**a**) MNPs, (**b**) MNPs_APTES, (**c**) MNPs_APTES_GA, (**d**) MNPs_APTES_-GA_BSA (0.2), (**e**) MNPs_APTES_GA_BSA (2). Accurate fit determined using χ^2^ and full width at half maximum of component peaks.

The XPS survey scan of MNPs before and after modification was conducted to analyze the chemical composition and surface changes shown in Figure 4. The findings support previous studies [40,41,42], indicating the absence of a satellite peak between Fe 2p1/2 and Fe 2p3/2 for Fe_3_O_4_. The peak positions for Fe 2p3/2 and Fe 2p1/2 were determined, and the observed peaks for Fe 3p3/2, O(1s), and C(1s) corresponded to the Fe-O bond characteristic of the Fe_3_O_4_ nanoparticles. The survey scan confirmed that silanization occurred due to the presence of nitrogen and silica peaks. The N 1s peak of APTES and further modified surfaces resulted from two overlapping peaks: NH_2_ and NH_3+_, and the Si 2p peak. Additionally, the high-resolution scan of the nitrogen peak indicated a high protonation level in the amine groups at the end of the APTES chain.

**Figure 5:**
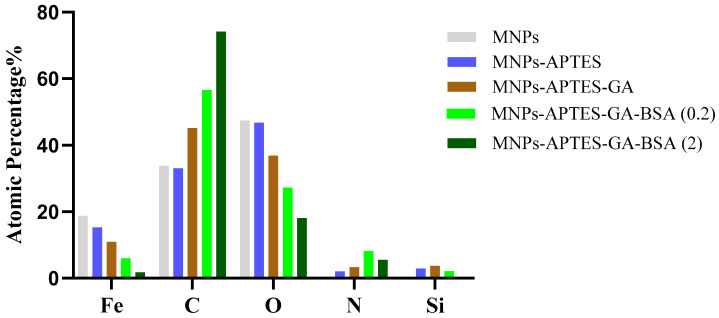
XPS chemical composition of MNPs, MNPs-APTES, MNPs-APTES-GA MNPs-APTES-GA-BSA (0.2 mg/mL), MNPs-APTES-GA-BSA (2 mg/mL). Accurate fit determined using χ^2^ and full width at half maximum of component peaks.

Furthermore, a significant change in the shape of the N 1s peak upon glutaraldehyde addition signified considerable chemical changes and the presence of new bonds. Also, the intensity shift for O 1s, C 1s, and Fe 2p through film formation further confirmed that the coating was successful. The chemical composition of samples shown in Figure 5 was determined using XPS analysis and the values confirmed the successful surface modification by APTES through the presence of nitrogen and silicon. Additionally, the atom % of iron decreased with each chemical addition, while the addition of albumin dramatically increased the relative amount of carbon in the sample, suggesting a thicker layer of albumin-crosslinked film was present.

### 2.4. Surface Charge Analysis

**Figure 6:**
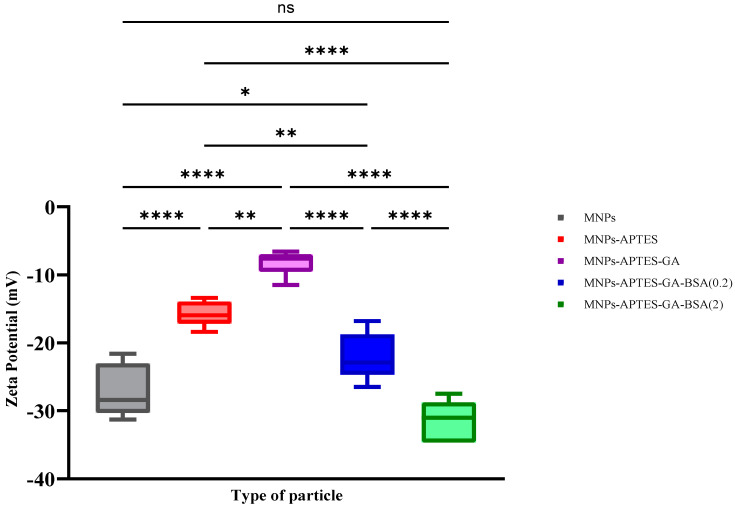
Zeta potential results for all MNP systems. Data are presented in box and whisker format, where the line in the box corresponds to the mean, and the whiskers represent the maximum and minimum of the data. * indicates *p*-values between 0.01 and 0.05, ** between 0.001 and 0.01, and **** for *p* < 0.0001, and “ns” means not statistically significant, data presented as mean ± 1 SD, n ≥ 3.

Zeta potential provides information on how surface modification affects solution-accessible surface charge, where zeta potentials greater than +30 mV or lower than −30 mV are generally indicative of long-term solution stability [43]. Zeta potential results for bare, APTES, APTES-GA, APTES-GA-BSA (0.2), and APTES-GA-BSA (2) MNP systems were similar to those in previous research on similar surfaces at −27.2 ± 3.8, −15.7 ± 1, −8.2 ± 2, −22.16 ± 3, and −31.36 ± 3 mV, respectively (Figure 6) [29,35,44,45,46].

The successful addition of APTES and glutaraldehyde was further confirmed through subsequent changes in surface charge [44]. Albumin incorporation caused a substantial decrease in zeta potential, as expected, given that albumin has a negative surface charge at this solution pH [36,47,48]. The higher albumin solution concentration led to a greater negative surface charge [49]. The *p*-value was calculated using an ordinary one-way ANOVA using GraphPad Prism 10.3 for all types of MNPs after every modification step.

### 2.5. Total Adsorbed Protein

**Figure 7:**
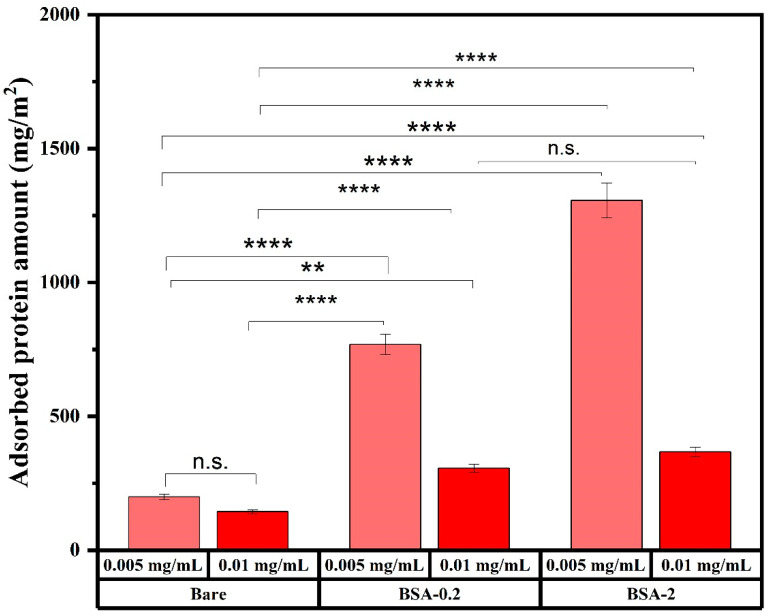
Representative results showing the total amount of plasma protein adsorbed to each MNP system, determined using BCA assay. ** Indicates *p* < 0.001 and **** *p* < 0.0001 and n.s. means not statistically significant, data presented as mean ± 1 SD, n ≥ 3.

To evaluate if the surface area of the MNP solution was saturated with adsorbed protein, two different MNP concentrations (0.005 and 0.01 mg/mL) were incubated in plasma and total adsorbed protein evaluated (Figure 7). Alb-MNP systems showed a significant reduction in adsorbed protein with increasing MNP concentration. BSA-2 and bare MNPs at 0.005 mg/mL showed the highest and lowest adsorbed protein amounts per unit area of MNP, respectively, and a similar trend was observed at higher MNP concentrations. In all cases, BSA-0.2 demonstrated a behavior between bare and BSA-2. Moreover, the amount of protein adsorbed by these MNPs was dependent upon BSA content. As we increased the BSA coating from 0, 0.2 to 2 mg/mL, the interaction behavior proceeded accordingly due to the uniform solubility of MNPs in plasma, which is consistent with the literature, as indicated by [50]. The statistical analysis was carried out using two-way ANOVA using GraphPad Prism 10.3. A *p*-value greater than 0.05 indicates no statistically significant difference in the means of the amounts for bare, BSA 0.2, and BSA 2 mg/mL MNPs at the two different concentrations.

### 2.6. Plasma Clotting in the Presence of BSA-Coated Nanoparticles

**Figure 8:**
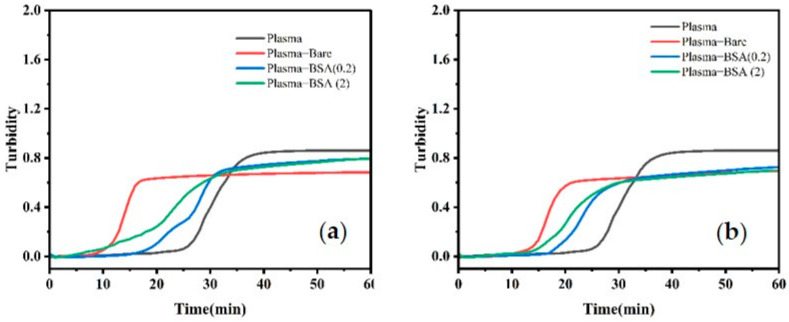
Presence of MNPs reduces clotting time driven by plasma proteins. Representative plots of baseline-corrected average clot formation in platelet-poor human plasma over 60 min, with bare particles, BSA (0.2), BSA 2.0 (**a**) for 0.005 and (**b**) for 0.01 mg/mL.

Contact activation studies for evaluating coagulation were conducted using bare and BSA-modified MNPs in concentrations of 0.005 and 0.01 mg/mL (Figure 8) [2] Many attributes of the surface are known to affect clotting time, including surface area and surface chemistry [51]. Introducing MNPs to the plasma significantly accelerated the clotting process compared to the control plasma sample [52]. Interestingly, this acceleration in clotting time was accompanied by a decrease in clot density (Table 1). Regardless of concentration, bare MNPs initiated a rapid clot formation with a similar slope but an onset time that was one-third of that of the control plasma. BSA-modified MNPs showed a reduced slope, a slower clot initiation time, and longer times to plateau compared to bare MNPs—despite these systems having similar charges. The values were observed to be within the normal range of 5 to 15 min. Surprisingly, increasing the concentration of BSA-MNPs led to a drastic delay in clot formation that was not observed for bare MNPs. The effective surface areas for bare and BSA modified particles are 452 ± 4, 803 ± 4, and 3177 ± 4 nm^2^.

### 2.7. Quantification of Plasma Protein Adsorption

Surface adsorption of plasma proteins was quantified using immunoblot band intensity and a 13-step grayscale system (Table 2) and images of Immunoblots available in (Supplementary file Figure S1). The role of each protein in plasma was extensively discussed by our team in prior publications [2,53].

Previous research using the immunoblotting technique showed that albumin has a strong affinity to BSA nanoparticles modified with poly-l-lysine and poly(ethylene glycol) [54]. This study also observed relatively high levels of albumin adsorption across all MNP systems, with bare MNPs reaching the maximum immunoblot intensity value at a concentration of 0.01 mg/mL. Compared to bare MNPs, BSA-2 MNPs at both low and high concentrations exhibited lower albumin adsorption. This reduced adsorption aligns with previous studies, where polysulfone membranes covalently modified with BSA demonstrated comparatively lower albumin adsorption than pristine membranes [55]. Similarly, polyethersulfone/poly(acrylonitrile-co-acrylic acid)-blended membranes, modified with BSA, exhibited lower albumin adsorption compared to non-modified polyethersulfone membranes [56].

#### 2.7.1. Immune-Response-Related Proteins

C3 plays a key role in complement activation, where the 42-kDa fragment is generated upon complement activation. Previous studies on BSA nanoparticles coated with poly-L-lysine and poly(ethylene glycol) demonstrated relatively high adsorption levels for the C3 β chain and the 42-kDa activation fragment, while the C3 α chain was not detected in those systems [54]. Here, the intact C3 was detected with very low to low adsorption levels observed on all MNP systems at both MNP concentrations. The minimum detectable intensity was observed in BSA-0.2 at the higher MNP concentration, while the highest intensity value among all systems for intact C3 (187 kDa) was observed in BSA-2 at 0.005 mg/mL concentration. All MNP systems at both concentrations showed relatively high band intensities for the α chain (115 kDa) of C3, reaching higher intensity levels for the β chain (70 kDa). BSA-2 at a 0.01 mg/mL concentration was the only system that showed very low intensity values for the α chain. The activation fragment was absent in almost all systems, with only a barely detectable band observed in the BSA-0.2 and BSA-2 systems, both at lower MNP concentrations. This suggests that the MNP systems are not actively involved in C3 activation. This is consistent with previous findings, where grafting albumin onto polyacrylonitrile membranes was found to induce lower complement activation compared to pristine membranes [57]. It has been suggested that BSA modification inhibits activation by blocking sites that could initiate complement activation [55].

IgG is involved in the activation of the classical complement pathway. Bare MNPs exhibited very high intensity values for both the heavy chain (55 kDa) and light chain (27 kDa) of IgG. BSA coating appeared to reduce IgG adsorption, with BSA-2 showing a noticeable decrease in band intensity at both concentrations for the 55-kDa heavy chain and 27-kDa light chain of IgG. A similar decreasing trend in band intensity was observed in the BSA-0.2 system for the 55-kDa heavy chain of IgG at both MNP concentrations and for the 27-kDa light chain of IgG at 0.005 mg/mL concentration.

Transferrin (77 kDa) is involved in macrophage activation. The intensity value for transferrin was consistently very high across all MNP systems at both concentrations, indicating that the binding of this protein may induce macrophage activity.

Vitronectin plays a role in the regulation of the complement system. Most MNP systems showed no band or minimal band intensity for vitronectin, with only BSA-0.2 exhibiting a slight increase in intensity for this protein at 0.005 mg/mL MNP concentration. While previous studies have demonstrated strong vitronectin adsorption to negatively charged polystyrene copolymer particles [58], immunoblot studies have shown that vitronectin was not detected on BSA nanoparticles [54].

α_1_-antitrypsin (54 kDa) is the most abundant serine proteinase inhibitor in plasma. Previous studies on iron oxide MNPs showed a moderate adsorption of α_1_-antitrypsin [59]. We previously observed relatively high adsorption levels of α_1_-antitrypsin on β-cyclodextrin-coated MNPs [2]. While BSA nanoparticles modified with poly-L-lysine and poly(ethylene glycol) have not shown adsorption of α_1_-antitrypsin [54], in this study, this protein was found to be adsorbed to MNP systems at varying levels. BSA-0.2 exhibited high adsorption levels of α_1_-antitrypsin at both MNP concentrations. BSA-2 exhibited a shift from high to low adsorption levels with increasing MNP concentration. Other MNP systems showed moderate adsorption levels of this protein.

α_2_-macroglobulin is a multifunctional protein with protease inhibitory properties in plasma, including clotting or fibrinolysis [60]. It has been observed to adsorb onto various surfaces, including elastin-like polypeptide nanoparticles [53], TiO_2_ nanoparticles [61], gold nanoparticles [62], and PEGylated liposomes [63], demonstrating its versatile interactions with different materials [64]. In previous immunoblot studies conducted by our team, no visible band was identified for this protease in MNPs coated with 2-(methacryloyloxy)ethyl phosphorylcholine [2]. All MNP systems at both low and high concentrations exhibited very low adsorption levels of this protein.

#### 2.7.2. Coagulation-Related Plasma Proteins

Fibrinogen (340 kDa) plays a key role in blood coagulation. Adsorption levels of fibrinogen, including all three chains (Aα—68 kDa, Bβ—56 kDa, and γ—48 kDa), were relatively high for all MNP systems at both high and low MNP concentrations. Notably, the BSA-2 system at a 0.01 mg/mL concentration exhibited a significant decrease in all fibrinogen chains compared to bare MNPs, with the γ-chain reaching very low band intensity. Fibrinogen cleavage fragments (<48 kDa) were not detected on most MNP systems, with only BSA-0.2 and BSA-2 both at a 0.005 mg/mL MNP concentration showing a very faint band. Studies have shown that covalent immobilization of albumin on the surface of polyacrylonitrile hemodialysis membranes reduces fibrinogen adsorption, inhibiting platelet adhesion and potentially improving hemocompatibility [57]. The relatively high level of fibrinogen is consistent with previous studies where PEG-coated BSA nanoparticles showed high adsorption levels of fibrinogen [54].

Prothrombin (72 kDa), the inactive precursor for thrombin, exhibited very low adsorption levels in most MNP systems. BSA-0.2 demonstrated a substantial increase in band intensity for prothrombin at both MNP concentrations compared to other MNP coatings. A similar trend in band intensity was observed for antithrombin, with BSA-0.2 at both MNP concentrations and BSA-2 at a lower MNP concentration reaching moderate band intensity levels. Previous studies have reported very low adsorption levels of prothrombin and antithrombin on 2-(methacryloyloxy)ethyl phosphorylcholine-coated MNPs [2], while no detectable bands were observed for these proteins on BSA nanoparticles coated with poly-l-lysine and poly(ethylene glycol) [54]. The impact of surface charge on prothrombin binding has been indicated by studies on different liposome formulations. These investigations have shown a tendency for prothrombin to bind more readily to cationic lipids such as DOTAP and DC-Cholesterol [65]. Substituting these cationic lipids with neutral ones has been found to decrease protein adsorption [65].

The coagulation contact pathway features three serine proteinases: Factors XII and XI, plasma prekallikrein (85 kDa), and high-molecular-weight kininogen as a cofactor [66]. For all MNP systems, Factor XII was not detected, and Factor XI exhibited consistent trace amounts across different MNP formulations, with only BSA-0.2 showing no band at 0.01 mg/mL concentration.

Plasminogen (91 kDa), the zymogen of plasmin, was found at very low to moderately low adsorption levels across all MNP systems [67]. While previous studies reported moderate plasminogen levels on poly-l-lysine-coated BSA nanoparticles [54], other research demonstrated increased plasminogen adsorption on polyurethane films modified with lysine as the surface lysine content increased.

Protein S, a key participant in coagulation regulation, was only detected in minimal quantities at low concentrations of BSA-0.2 and in both high and low MNP concentrations of BSA-2. This protein was not detected on other surfaces, including poly-l-lysine/poly(ethylene glycol)-coated BSA nanoparticles, bare and β-cyclodextrin coated MNP [2,54]. The high-molecular-weight kininogen, low-molecular-weight kininogen, Factor I, prekallikrein, fibronectin, and protein C were screened without detection even in trace amounts for any of the MNP systems.

## 3. Materials and Methods

### 3.1. Materials

Sodium hydroxide (Fisher Scientific, Hampton, NH, USA), FeCl_2_·4H_2_O, FeCl_3_·6H_2_O, ammonium hydroxide solution (25%), Aminopropyltriethoxysilane (APTES, 99%), and BSA (98%) were purchased from Millipore Sigma , Oakville, ON, Canada. Absolute ethanol (99.5%) and phosphate-buffered saline (PBS) in HPLC-grade water (0.01 M, pH 7.4) were purchased from Fisher Bioreagents tablets (filtered, 0.22 µm), Glutaraldehyde 70% EM-grade was purchased from Electron Microscopy Sciences, Hatfield, PA, USA. Sodium phosphate dibasic heptahydrate, sodium phosphate monobasic monohydrate, and PBS tablet were purchased from Fisher Scientific Hampton, NH, USA. Platelet-poor human plasma was obtained from the Blood4Research program from Canadian Blood Services. Sodium dodecyl sulfate (SDS) and polyvinylidene fluoride (PVDF) membrane were used (Bio-Rad, Hercules, CA, USA). TMB-stabilized substrate (Promega, Madi-son, WI, USA) was used. BCA protein assay was used (Pierce™ BCA Protein Assay Kit, Thermo Fisher Scientific Inc., Waltham, MA, USA). For the full list of antibodies, see Table S1 in the Supplementary Materials. Statistical analysis was conducted using a Student’s *t*-test.

### 3.2. Synthesis of Magnetite (Fe_3_O_4_) Iron Oxide Nanoparticles

A three-necked round bottom flask was filled with N_2_, FeCl_2_·4H_2_O (1 g, 5.0 mmol) and FeCl_3_·6H_2_O (2.6 g, 9.6 mmol) dissolved in 25 mL degassed water, and stirred at 400 rpm (75 °C) for 10 min; 10 mL of ammonium hydroxide solution (25%) was added slowly, and stirring increased to 600 rpm. The reaction was terminated after stirring for 1.5 h. The resulting nanoparticles were washed with water and ethanol (99.5%) thrice, magnetically separated, and vacuum-dried.

### 3.3. Amino-Silane Surface Treatment

The surface treatment of the iron oxide MNPs was carried out using a slightly modified protocol [68]. Briefly, 50 mg of Fe_3_O_4_ MNPs was synthesized by the above-mentioned procedure, suspended in 5 mL of absolute ethanol (99.5%), vortexed, and sonicated for 10 min. A nitrogen atmosphere was established over the suspension and held for 5 min under gentle stirring; 35 µL of APTES was added, and the nitrogen atmosphere was re-established, and left overnight at room temperature to react under gentle stirring. Particles were washed twice with absolute ethanol and twice with deionized water (0.22 µm filtered), suspended in 5 mL of PBS, and 0.6 mL of 70% glutaraldehyde was added to the suspension. The solution was stirred for 3 h at room temperature, and the particles washed thrice with normal PBS using HPLC-grade water (0.01 M, pH 7.4), suspended in PBS, and stored at 4 °C until further use.

### 3.4. Albumin Coating Formation

Alb-MNPs were prepared with final reaction concentrations of 0.08 mg/mL of MNPs and solutions of 0.2 or 2 mg/mL of albumin, as previously documented [33,69]. Briefly, BSA was dissolved in half the final volume of PBS for the reaction, and MNPs were suspended in the other half and sonicated. These solutions were combined in a round bottom flask, and the reaction proceeded at room temperature in a shaker at 150 rpm for 2 h. After reaction with BSA, the particles were washed several times with PBS and suspended in PBS at 4 °C until further use.

### 3.5. Particle Characterization

Particle size and morphology were characterized using TEM (JEM-ARM200CF S/TEM, JEOL, Houston, TX, USA) at an accelerating voltage of 200 kV and LaB6 filament. To prepare TEM samples, 5 µL highly diluted samples of MNPs were spotted on a carbon grid and dried in a vacuum overnight. The TGA analysis was conducted to determine the thermal stability of both modified and unmodified MNP samples. Freeze-dried powder of each sample was prepared, and TGA was conducted (Pyris 1 TGA, Perkin Elmer, Waltham, MA, USA) with a temperature ramp of 10 °C/min from 25–800 °C under flowing nitrogen. A 1.39 mg sample was used for MNPs, 0.62 mg for MNP-APTES, 0.39 mg for MNP-APTES-GA, 0.33 mg for MNP-APTES-GA-BSA (0.2), and 0.55 mg for MNP-APTES-GA-BSA (2). Particle zeta potential was measured (Zetasizer, Nano ZS, Malvern Instruments, Malvern, UK) using 25 µL of 1 mg/mL MNPs suspension in 3 mL DI water at pH 7, similar to blood. The average zeta potential value was obtained using three repeats. Particle surface chemistry was analyzed using XPS (Kratos Ultra XPS Spectrometer, Manchester, UK) using 1 mg of freeze-dried powder for each sample.

### 3.6. Immunoblot Techniques

Human plasma experiments were conducted and followed the guidelines of the research ethics board approvals from Canadian Blood Services 2022–21 and University of Alberta Pro00002363 and Pro00116764. Incubating MNPs with platelet-poor plasma was conducted according to established procedures [53]. Briefly, a 0.25 mL solution of different MNPs (i.e., Bare MNPs, MNPs-APTES-GA-BSA (0.2), MNPs-APTES-GA-BSA (2)) prepared in 10 mM PBS was added to 1 mL of plasma at 37 °C at the same concentration used for recalcification turbidimetric assay (i.e., 0.005 and 0.01 mg/mL) and incubated at 37 °C, 18 rpm for 2 h in vertical rotating direction. After 2 h of incubation, the samples were centrifuged at 20,000× *g* for 10 min. The supernatant was removed, and MNPs were washed twice with 1 mL of PBS to remove loosely bound proteins. This process was carried out by removing the plasma from the tubes and adding fresh 1 mL of 10 mM PBS pH 7.4 into the tubes every time and incubating for 30 min at 37 °C. After 30 min, these were centrifuged at 20,000× *g* for 10 min at room temperature. PBS incubation was carried out twice using fresh PBS each time. The final pellet of particles and adsorbed proteins were resuspended in 100 μL of 10% SDS in PBS and incubated at 50 °C for 2 h, then centrifuged at 21,000 rpm for 10 min to elute the adsorbed proteins from the surface of MNPs. Eluted protein sample concentration was quantified using the Pierce™ BCA protein (detergent-compatible) assay. The protein profiling of eluted samples was compared using SDS-PAGE and immunoblot techniques, following a previously established protocol [53,60]. Briefly, upon running equal volumes of eluted sample (100 mL), the gels were transferred to polyvinylidene difluoride membranes. The membranes were vertically cut into 2 mm wide strips, each utilized for immunoblotting with a different type of primary antibody (1:1000 dilution). For visualization, HRP-conjugated secondary antibodies with TMB substrate were used. To ensure intensity comparability, a consistent 10 min color development process was applied to all immunoblots.

### 3.7. MNP Incubation with Plasma

Clot formation was assessed using plasma calcification turbidimetric assay. Plasma (100 μL) was incubated with 25 μL of different types of MNPs in PBS solution. Plasma was incubated with PBS (10 mM) for 30 min, then incubated with MNPs for 1 h prior to testing. Then, 100 μL of 0.025 M CaCl_2_ was injected into a 96-well plate for turbidity reading. A BioTek ELx808 plate reader was used to measure the absorbance at 405 nm at 1 min intervals over a 60 min period. All steps were performed at 37 °C with three independent repeats.

### 3.8. Statistical Analysis

Statistical analysis was conducted by analyzing the zeta potential of MNPs after every modification step and quantification of immunoblot data from three independent experiments to assess the total adsorbed amount for all types of MNPs (bare and BSA-modified particles). Statistical analysis was conducted using a two-way ANOVA, considering unequal variances between the samples. Calculated *p*-values were defined as *p* < 0.01 and *p* < 0.05. Non-significant differences are labeled as “ns”.

## 4. Conclusions

The hemocompatibility of modified and unmodified iron oxide magnetic nanoparticles (MNPs) was studied to assess the influence of surface properties on their interaction with plasma. A comprehensive physicochemical characterization was conducted using transmission electron microscopy (TEM), zeta potential, and thermogravimetric analysis (TGA). This was followed by bicinchoninic acid (BCA) Assay, Sodium Dodecyl Sulphate-Polyacrylamide Gel Electrophoresis (SDS-PAGE), and immunoblotting to evaluate the adsorption of plasma proteins. The clotting parameters were assessed using both modified and unmodified iron oxide nanoparticles by interacting with platelet-poor plasma to observe the time point of clot formation. The TEM, TGA, and zeta potential results confirm the coating of protein on bare iron oxide nanoparticles and effectively change the attributes of iron oxide nanoparticles. Upon increasing the concentration of iron oxide nanoparticles (both bare and modified), we noted a decrease in protein adsorption. A significant reduction in the amount of adsorbed protein was noted when comparing bare iron oxide nanoparticles (MNPs) to BSA-modified iron oxide nanoparticles, being highest for BSA-2 iron oxide nanoparticles. It seems plasma proteins are primarily attracted by the surface chemistry of the iron oxide nanoparticles. Introducing iron oxide magnetic nanoparticles (MNPs) to plasma resulted in a markedly faster clotting process compared to a standard plasma sample. This increase in clotting speed was associated with a reduced density of the clots formed. Irrespective of their concentration, iron oxide magnetic nanoparticles (MNPs) without any modification caused quick clot formation, displaying a similar rate of clot formation but initiating approximately three times faster than the control plasma sample. BSA-modified iron oxide magnetic nanoparticles (MNPs) exhibited a decrease in clot formation rate, took longer to begin forming clots, and required more time to reach their maximum clotting capacity when compared to the unmodified iron oxide magnetic nanoparticles (MNPs) despite both types of iron oxide nanoparticles carrying similar electrical charges. Immunoblots show that modified iron oxide nanoparticles showed changes in albumin, protein C, IgG, transferrin, fibrinogen, α1-antitrypsin, vitronectin, plasminogen, prothrombin, and antithrombin levels compared to unmodified controls. The safe use of iron oxide magnetic nanoparticles for blood-based applications seems to require surface modification and albumin affects the hemocompatibility of the surface.

## Figures and Tables

**Table 1:** Summary of platelet-poor plasma clotting initiation and completion time of bare, BSA-0.2 and BSA-2 magnetic nanoparticles (n = 3, all SDs are less than fifteen seconds).

		Plasma	Bare MNPs	BSA-0.2	BSA-2
0.005 mg/mL	Turbidity	0.85	0.70	0.72	0.69
	Clotting Starting point (min)	18	6	9	6
	Plateau reach point (min)	39	18	30	34
0.01 mg/mL	Turbidity	0.85	0.72	0.69	0.65
	Clotting Starting point (min)	18	7	17	14
	Plateau reach point (min)	39	21	32	34

**Table 2:** Relative intensities of the immunoblot of plasma proteins adsorbed to different types of MNP systems ranging from absence of a band (0) to maximum intensity (12) on the grayscale. The intermediate levels indicate various degrees of intensity: 1–3 (very low), 4–5 (relatively low), 6–7 (moderate), 8–9 (relatively high), 10–11 (high), and 12 (very high).

Plasma Proteins				Bare		BSA-0.2		BSA-2		
			Fragment Size (kDa)	0.005 (mg/mL)	0.01 (mg/mL)	0.005 (mg/mL)	0.01 (mg/mL)	0.005 (mg/mL)	0.01 (mg/mL)	
	Albumin		66	10	12	9	10	9	8	0
Immune response-related	C3	Whole C3	187	4	2	3	1	5	3	1–3
		α chain	115	9	7	9	9	7	3	4–5
		β chain	70	11	11	10	10	9	8	6–7
		Activation fragment	42	0	0	1	0	1	0	8–9
	IgG	Heavy chain	55	10	10	7	7	8	6	10–11
		light chain	27	10	11	9	11	7	8	12
	Transferrin		77	11	11	11	11	10	10	
	Vitronectin		54	0	0	4	1	2	1	
	α 1 antitrypsin		54	5	6	10	10	10	7	
	α 2 macroglobulin		163	1	2	3	1	2	1	
	Protein S		75	0	0	1	0	1	1	
Coagulation related	Fibrinogen	α chain	68	8	8	8	8	8	6	
		β chain	56	9	10	7	7	9	5	
		γ chain	48	7	8	7	7	7	3	
		Cleavage fragments	<48	0	0	1	0	1	0	
	Prothrombin		72	2	1	5	6	3	0	
	Antithrombin		53	2	3	7	5	6	0	
	Factor XII		80	0	0	0	0	0	0	
	Factor XI		70	1	1	1	0	1	1	
	Prekallikrein		85	0	0	0	0	0	0	
	Protein C		62	0	0	0	0	0	0	
	Plasminogen		91	4	1	3	4	3	3	

## Data Availability

The data used to support the findings of this study are included in the article and Supplementary Materials.

## Supplementary Materials

The following supporting information can be downloaded at: https://www.mdpi.com/article/10.3390/ijms252211975/s1.
