# Supplementary material for: Hemocompatibility of Albumin-Modified Magnetic Nanoparticles
Source: Int J Mol Sci. 2024 Nov 7;25(22):11975. doi: 10.3390/ijms252211975 (PMC11593459; doi:10.3390/ijms252211975)
Supplement: Supplementary file 1 [file ijms-25-11975-s001.zip › ijms-3268048-supplementary.pdf]

Supplementary Information for Hemocompatibility of Albumin-Modified Magnetic Nanoparticles

Indu Sharma, Mehdi Gaffari Sharaf, Aishwarya Pawar, Agatha Milley and Larry D. Unsworth *

Department of Chemical and Material Engineering, University of Alberta, Edmonton, AB T6G 1H9, Canada; indu2@ualberta.ca (I.S.); mghaffar@ualberta.ca (M.G.S.); aspawar@ualberta.ca (A.P.); amilley@ualberta.ca (A.M.)

* Correspondence: lunswort@ualberta.ca; Tel.: +1-780-492-6020; Fax: +1780-492-2881

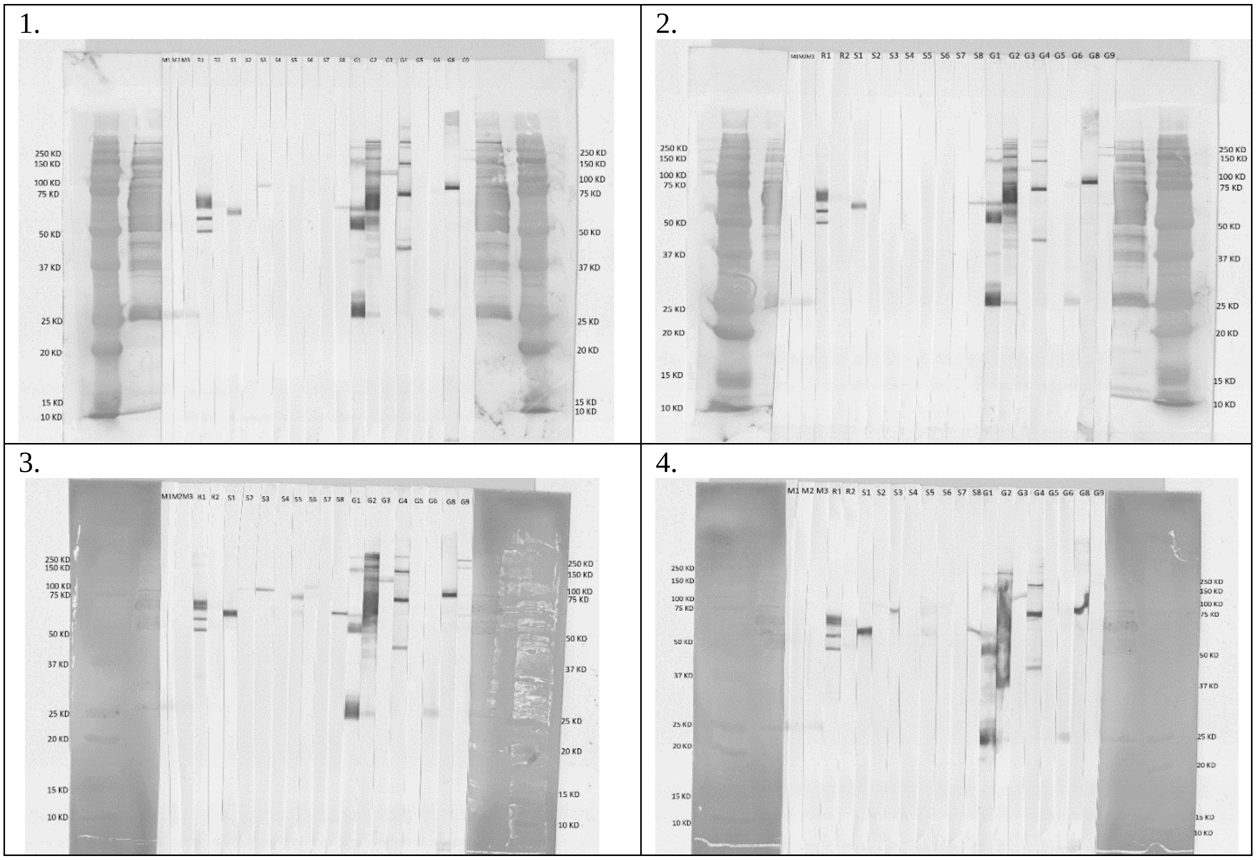

5.

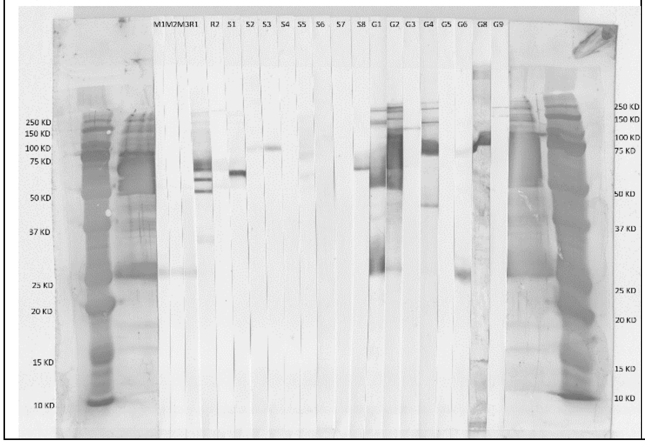

6.

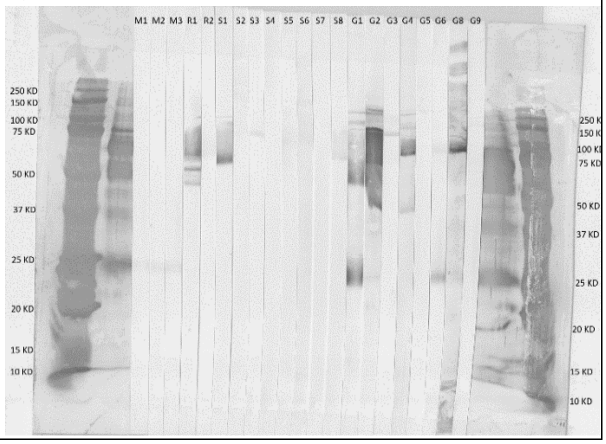

Figure S1. Representative reassembled western blot membrane of eluted plasma proteins (1-2) bare MNPs, (3-4) Particle BSA (0.2), (5-6) Particle BSA (2). The middle strips (1-21) are processed in western blots, and two side pieces are non-specifically stained with colloidal gold stain.

I	Ladder
II	Protein sample stained with non-specific colloidal gold
1	Ani-kininogen (light)
2	Ani-kininogen (heavy)
3	Anti-factor I
4	Anti-fibrinogen α, β, and γ
5	Anti-fibronectin
6	Anti-Alpha1 antitrypsin
7	Anti-thrombin
8	Anti-prothrombin
9	Anti-protein C
10	Anti-vitronectin
11	Anti-protein S
12	Anti-Prekallikrein
13	Anti-antithrombin
14	Anti-IgG
15	Anti-human albumin
16	Anti-plasminogen
17	Anti-C3
18	Anti-factor XII
19	Anti-factor XI
20	Anti-transferrin
21	Anti-alpha2 macroglobulin

Table S1. Primary antibodies against human plasma proteins used in immunoblot studies.

Anti-Human Plasma Antibody	Host	Vendor
Albumin	Goat	OEM Concepts, Saco, ME, USA
Antithrombin	Sheep	Cedarlane Laboratories, Hornby, Ontario, Canada
Complement factor 3	Goat	Calbiochem, Gibbstown, NJ, USA
Factor I	Mouse	Invitrogen; Thermo Fisher Scientific Inc.
Factor XI	Goat	Cedarlane Laboratories, Hornby, Ontario, Canada
Factor XII	Goat	Cedarlane Laboratories, Hornby, Ontario, Canada
Fibrinogen	Rabbit	Calbiochem, Gibbstown, NJ, USA
Fibronectin	Rabbit	Cedarlane Laboratories, Hornby, Ontario, Canada
IgG	Goat	Sigma-Aldrich, St. Louis, MO, USA
Kininogen (heavy chain)	Mouse	US Biological, Swampscott, MA, USA
Kininogen (light chain)	Mouse	US Biological, Swampscott, MA, USA
Plasminogen	Goat	Cedarlane Laboratories, Hornby, Ontario, Canada
Prekallikrein	Sheep	Cedarlane Laboratories, Hornby, Ontario, Canada
Protein C	Sheep	Cedarlane Laboratories, Hornby, Ontario, Canada
Protein S	Sheep	Cedarlane Laboratories, Hornby, Ontario, Canada
Prothrombin	Sheep	Cedarlane Laboratories, Hornby, Ontario, Canada
Thrombin	Sheep	Cedarlane Laboratories, Hornby, Ontario, Canada
Transferrin	Goat	Sigma-Aldrich, St. Louis, MO, USA
Vitronectin	Sheep	Cedarlane Laboratories, Hornby, Ontario, Canada
α1-Antitrypsin	Sheep	Cedarlane Laboratories, Hornby, Ontario, Canada
α2-Macroglobulin	Goat	Sigma-Aldrich, St. Louis, MO, USA
